# Assessment of medical waste management in seven hospitals in Lagos, Nigeria

**DOI:** 10.1186/s12889-016-2916-1

**Published:** 2016-03-15

**Authors:** Olufunsho Awodele, Aishat Abiodun Adewoye, Azuka Cyril Oparah

**Affiliations:** Department of Pharmacology, Therapeutics and Toxicology, College of Medicine, University of Lagos, PMB 12003 Lagos, Nigeria; Transnational Environmental Co., 75, Tejuosho Surulere, Yaba, Lagos Nigeria; Department of Clinical Pharmacy and Pharmacy Practice, University of Benin, Benin City, Edo State Nigeria

**Keywords:** Medical waste, Waste management, Healthcare workers, Environmental hazards, LAWMA and Lagos

## Abstract

**Background:**

Medical waste (MW) can be generated in hospitals, clinics and places where diagnosis and treatment are conducted. The management of these wastes is an issue of great concern and importance in view of potential public health risks associated with such wastes. The study assessed the medical waste management practices in selected hospitals and also determined the impact of Lagos Waste Management Authority (LAWMA) intervention programs. A descriptive cross-sectional survey method was used.

**Methods:**

Data were collected using three instrument (questionnaire, site visitation and in –depth interview). Two public (hospital A, B) and five private (hospital C, D, E, F and G) which provide services for low, middle and high income earners were used. Data analysis was done with SPSS version 20. Chi-squared test was used to determine level of significance at *p* < 0.05.

**Results:**

The majority 56 (53.3 %) of the respondents were females with mean age of 35.46 (±1.66) years. The hospital surveyed, except hospital D, disposes both general and medical waste separately. All the facilities have the same process of managing their waste which is segregation, collection/on-site transportation, on-site storage and off–site transportation. Staff responsible for collecting medical waste use**s** mainly hand gloves as personal protective equipment. The intervention programs helped to ensure compliance and safety of the processes; all the hospitals employ the services of LAWMA for final waste disposal and treatment. Only hospital B offered on-site treatment of its waste (sharps only) with an incinerator while LAWMA uses hydroclave to treat its wastes. There are no policies or guidelines in all investigated hospitals for managing waste.

**Conclusions:**

An awareness of proper waste management amongst health workers has been created in most hospitals through the initiative of LAWMA. However, hospital D still mixes municipal and hazardous wastes. The treatment of waste is generally done by LAWMA using hydroclave, to prevent environmental hazards except hospital B that treats its sharp with an incinerator. In order to enhance uniform and appropriate waste management practices in the entire State, there is need for capacity building at all levels and also policies and guidelines formulations.

## Background

Medical waste management (MWM) has become a critical issue as it poses potential health risks and damage to the environment [1, 2]. It is also of greater importance due to its potential environmental hazards and public health risks with high propensity to result into epidemics [3].

It continues to be a major challenge, particularly, in most healthcare facilities of the developing countries where it is hampered by technological, economical, social difficulties and inadequate training of staff responsible for handling of the waste [4]. Poor conduct and inappropriate management and disposal methods exercised during handling and disposal of medical waste (MW) is an increasing significant health hazards and environmental pollution/hazards due to the infectious nature and unpleasant smell of the waste [5–7]. Despite the fact that current medical waste management (MWM) practices vary from hospital to hospital, the problematic areas are similar for all healthcare units and at all stages of management [8].

In Nigeria, a typical developing African nation, not many people are aware that medical waste contributes substantially to environmental pollution and hazards. This is reflected by lack of awareness and specific policy to address the menace of healthcare facility (HCF) waste, some of which is deemed hazardous [9]. It is important to note that healthcare wastes, if not properly managed, could pose an even greater threat and hazards than the original diseases. It is the duty of hospital and healthcare centers to take care of public health issues such as MW. Specific approaches that may be employed include patient care and enlightenment, ensure clean and healthy environment for workers/community [10]. Carefree handling and disposal of MW impacts both directly and indirectly on staff, patient and environment. This is because the hospitals represent a unique environment, providing healthcare to patients and work environment for medical and other staff.

In the process of healthcare delivery, medical waste is generated, which includes sharps, human tissues or body parts and other infectious materials [11]. Interestingly, there are reasonable ranges of technologies available for the treatment of healthcare wastes that may be appropriate for use in the third world countries.

The World Health Organization (WHO) estimates that each year there are about 8 to 16 million new cases of Hepatitis B virus (HBV), 2.3–4.7 million cases of Hepatitis C virus (HCV) and 80,000–160,000 cases of Human Immunodeficiency Virus (HIV) due to unsafe injections disposal and mostly due to very poor waste management systems [12].

Contaminated injection equipment may be scavenged from waste areas and dump site either to be reused or sold to be used again. The negative health and environmental impacts of MW includes transmission of diseases by virus and microorganism, defacing the aesthetics’ of the environment, as well as contamination of underground water tables by untreated MW in landfills [13]. Good medical waste management in hospital depends on a dedicated waste management team, good administration, careful planning, sound organization, underpinning legislation, adequate financing and full participation by trained staff [14].

However, it is pertinent that before any of these options is adopted, hospitals and medical facilities will need to assess the problems and put forward a management strategy that is suitable to their economic circumstances and also sustainable for use, based on local technology [15, 16]. Paradoxically, health-care activities which are meant to protect health, cure patients and save lives have been known to also generate waste. About 20 % of these wastes pose high risk, either of infection and chemical or radiation exposure [17].

Health-care activities generate significant amounts of hazardous waste such as mercury and expired pharmaceuticals, as well as large amounts of general waste. As a matter of fact, the management of health-care waste is an integral part of a national health-care system. A holistic approach to health-care waste management should include a clear delineation of responsibilities, occupational health and safety programs, waste minimization and segregation, development, adoption of safe and environmentally sound technologies, and capacity building.

Recognizing the urgency of this problem, a growing number of countries have taken initial steps to respond to this need. These include the establishment of regulatory frameworks, development of national plans and the demonstration of innovative approaches. However, funding of health-care waste management remains very inadequate [18].

This is an issue taking central place in the national health policies of many countries however, in most urban areas in Nigeria there are often no systematic approaches to MWM and it has not received sufficient attention. This may be because very often, health issues compete with other sectors of the economy for the very limited resources available. Also, in many countries, medical wastes are still handled and disposed together with domestic wastes, posing a great health risk to municipal workers, the public and the environment [19, 20]. Medical waste must be separated from municipal waste, but in many parts of Africa it tends to be collected along with the rest of the waste stream [20–22]. Furthermore, hospital wastes are still mixed with the municipal waste in collecting bins at roadsides and disposed of similarly [15, 23].

**Fig. 1:**
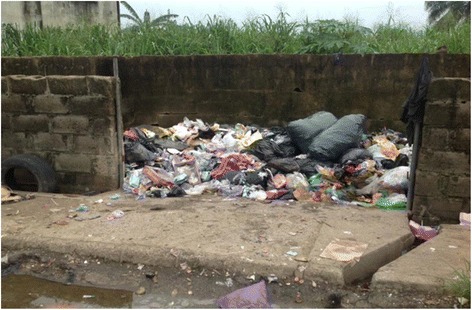
Improper storage of both general waste and infectious waste at one of the hospitals surveyed

In Korea, medical waste was often mixed with municipal solid waste and disposed of in residential waste landfills or improper treatment facilities (e.g. inadequately controlled incinerators) [24]. This is also evident as some of the hospital surveyed in Lagos mixes municipal and medical waste in their on – site storage facility (Fig. 1).

**Fig. 2:**
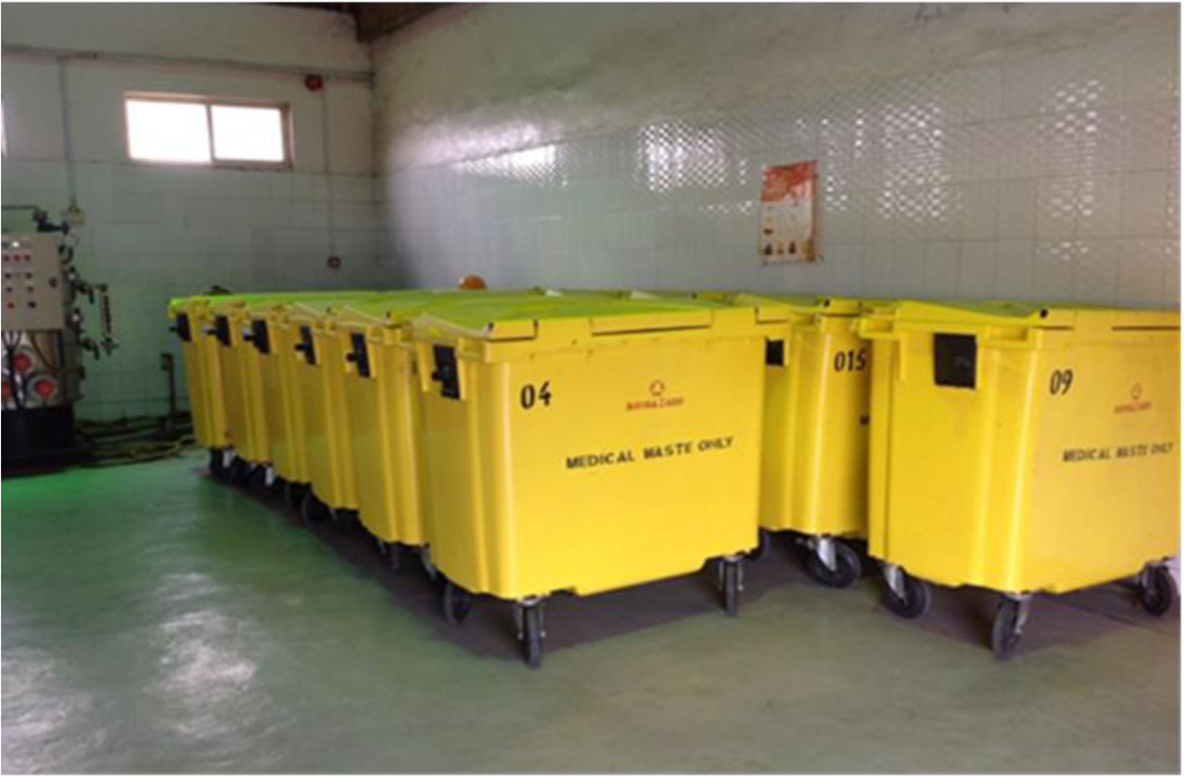
Lagos State special containers for loading medical waste at a transfer loading station

The population of Lagos State is on the increase and the amount of hospital waste generated is snowballing at alarming rates due to growth of population and healthcare facilities. However, there are some problems encountered with the management of MW and they are- improper storage, frequent dumping of infectious waste with municipal waste, no uniform definition and identification of hazardous waste and low level of awareness about the management of medical waste. It is worthy to note that Lagos State has gone a step ahead of federal government of Nigeria in the management of medical waste because of their intervention programs and also the construction of several well-equipped transfer loading stations available in some parts of the State (Fig. 2).

Therefore, this present study assessed the medical waste management practices in selected hospitals in Lagos State and also determined the impact of Lagos Waste Management Authority (LAWMA) intervention programs on medical waste management in Lagos, Nigeria.

## Methods

### Design & setting

The study employed an observational cross-sectional design conducted in Surulere, Mushin/Yaba, Ikeja, Gbagada and Lagos Island areas of Lagos State. Lagos is located in south western Nigeria on the western coast of Africa. Lagos is the most populous city in Nigeria, the largest country in Africa. The metropolitan area has an estimated 300 km^2^, a group of islands endowed with creeks and a lagoon. Officially, the population of Lagos was last recorded at 7,937,932 (2006 Census). Lagos is the second fastest growing city in Africa and the seventh fastest in the world. The population is an estimated 21 million (2011) which is 10 % of Nigeria’s population, recently projected at 167 million by the National Population Commission. (Punch Newspaper- November 20, 2011). Healthcare facilities are dispersed all over the metropolis and wastes generated from these facilities are often mixed with municipal waste.

### Study population

The target population of this survey consisted of selected 120 personnel (doctors, nurses, laboratory scientists and domestic workers from both private and public hospitals) in Lagos, Nigeria.

### Selection of facilities

Seven (7) hospitals were selected for the exercise, using stratified, simple random and convenience sampling methods. The hospitals were stratified into private and public based on the ownership of the hospitals. This approach ensured that the various categories of hospitals operating in Lagos were included in the study and coding of the hospitals was done to ensure anonymity/confidentiality.

The studied hospitals provide general medical, surgical, pediatric, maternity and a range of specialist services. The two (2) selected public hospitals include the only federal teaching hospital in Lagos State and one out of the twenty six (26) general hospitals owned by Lagos state. Five (5) private hospitals were also selected out of the nine hundred (900) private hospitals in Lagos using both simple random and convenience sampling methods. The hospitals were coded A, B, C, D, E, F and G. The two public hospitals (A and B) are among the largest and leading healthcare institutions in Lagos and, indeed, the oldest and most advanced facilities in Lagos State. The selected private hospitals serve the low-income, middle-income and high-income earners in Lagos State.

### Data collection

A catalog of the waste generated in each of the sampled hospital in the study area was carried out. The type of waste generated was identified through direct surveillance (site visitation) and use of questionnaire (sections of the questionnaire are; demographic Information, description of hospital, knowledge about the waste characterization, assessment of medical waste management practice, Information about the personnel involved in the management of waste, hospital waste management policy). In addition, the head of nurses, sanitary workers and laboratory officers were verbally interviewed with a view to obtaining the level of training of its staff. In each hospital, the questionnaires were administered to the doctors, nurses, laboratory officers and domestic workers/cleaners who were randomly selected for this purpose based on the proportion of staff in each hospital (see Table 1).

**Table 1:** Socio-demographic Characteristics of Respondents

Variable	No Participants/Frequency	Percentage
Type of facility		
A	23	21.9
B	41	39.0
C	9	8.6
D	4	3.8
E	11	10.5
F	7	6.7
G	10	9.5
Total	105	100.0
Age of respondents (years)		
20–25	9	8.6
26–30	18	17.1
31–35	26	24.8
36–40	12	11.4
41–45	20	19.0
> 45	15	14.3
Non response	5	4.8
Total	105	100.0
Religion of respondents		
Christian	75	71.4
Muslim	16	15.2
Non response	14	13.3
Total	105	100.0
Sex of respondents		
Male	42	40.0
Female	56	53.3
Not indicated	7	6.7
Total	105	100.0
Duration of working in the hospital		
1–5 years	35	33.3
6–10 years	30	28.6
11–15 years	19	18.1
16–20 years	8	7.6
> 20 years	10	9.5
Non response	3	2.9
Total	105	100.0
Profession of respondents		
Doctors	12	11.4
Nurses	33	31.4
Lab scientists	11	10.5
Domestic workers	36	34.3
Others	4	3.8
Non response	9	8.6
Total	105	100.0

The method adopted for this study follows the procedure used by Longe and Williams [25]. This involves the three instruments which are Survey questionnaire administration, Site visitation and in – depth interview. There were no existing waste management policy with respect to waste generation, segregation, collection, storage, transportation and final disposal in the hospitals however; a procedure was followed due to the training received from LAWMA/John Snow Inc.

### Analysis

Statistical Package for Social Sciences (SPSS version 20) was used for the analysis of the data. Chi-Square statistical test of significance was used to determine the level of significance of association between variables at 95 % confidence level (±5 % sampling error). Level of significance was set at *p* ≤ 0.05.

### Ethical consideration and participants consent

Ethical approval for this study was obtained from Lagos State Ministry of Health thereafter, institutions Health Research and Ethics Committee (HREC) approval was obtained. The experimental procedures were explained to the individual participants and thereafter their consent to participate in the study was obtained. The participants that declined not to be part of the study were excluded. Confidentiality was assured by excluding all the names of the hospital surveyed.

## Results

One hundred and five (105) questionnaires were fully completed out of the 120 questionnaires distributed in this study, giving a response rate of 87.5 %. The mean age of respondents was 35.46 ± 1.66 years.; majority of them were females 56 (53.3 %). The mean number of years spent in the hospital by respondents is 9.73 ± 6.91 year. The majority of respondents were domestic workers (34.3 %) and nurses (31.4 %) (Table 1).

The survey indicates that, apart from hospitals D and G, others have records of the volume of waste which they generate. The medical wastes generated range from 0.116 to 0.561 kg/bed/day, while the total waste is about 215.56 kg/day. Thus, the average generation rate is approximately 0.181 kg/bed/day.

The various categories of waste; general, pathological, chemical, infectious, sharp and pharmaceutical were found in all the hospital units, apart from the Pharmacy which does not generate pathological waste, the laundry, kitchen, administration and engineering units also generate general wastes alone (Table 2).

**Table 2:** Total types of medical wastes generated from the seven hospitals

Units	General (%)	Pathological (%)	Chemical (%)	Infectious (%)	Sharp (%)	Pharmaceutical (%)
Medical	65 (61.9)	15 (14.3)	23 (61.9)	45 (42.9)	12 (11.4)	3 (2.9)
Surgical	78 (74.3)	56 (53.3)	19 (18.1)	28 (26.7)	42 (40.0)	17 (16.2)
Operation	52 (49.5)	11 (10.5)	21 (11.4)	29 (27.6)	38 (36.7)	13 (12.4)
Dialysis	47 (44.8)	24 (22.9)	29 (27.6)	39 (37.1)	26 (24.8)	23 (21.9)
Oncology	67 (63.8)	22 (21.0)	2 (2.9)	38 (36.2)	26 (24.8)	6 (5.7)
Emergency	88 (83.8)	19 (18.1)	29 (27.6)	31 (29.5)	1 (1.0)	16 (15.3)
Radiology	3 (2.9)	65 (61.9)	48 (45.7)	30 (28.6)	22 (21.0)	14 (13.3)
Pathology	35 (7.0)	71 (67.6)	10 (9.2)	39 (37.1)	13 (12.4)	22 (21.0)
Biochemistry	62 (59.0)	25 (23.8)	34 (32.4)	47 (37.1)	13 (12.4)	22 (21.0)
Microbiology	59 (56.1)	18 (17.1)	14 (13.3)	49 (37.9)	28 (26.7)	15 (14.3)
Blood bank	58 (44.8)	15 (14.3)	13 (12.4)	35 (33.3)	34 (32.4)	16 (15.2)
Pharmacy	49 (46.7)	None	18 (17.1)	14 (13.3)	12 (11.4)	21 (20.0)
Laundry	54 (51.4)	None	None	None	None	None
Kitchen	56 (53.3)	None	None	None	None	None
Administration	47 (44.7)	None	None	None	None	None
Engineering	49 (44.8)	None	None	None	None	None

The respondents in the various facilities had adequate knowledge of waste categorization. About 69.5 % of the respondents rightly categorized paper, food, plastics and bottles as general waste. Soiled cotton wool, swab and gloves were also classified by 69.5 % of the respondents as infectious wastes. The majority of respondents also got it right by classifying body parts, body fluids and fetuses as pathological wastes (Table 3). There was a significant association (*p* < 0.05) between the profession of the respondents and categorization of paper, bottles, food and plastic wastes. However, there were no significant differences (*p* > 0.05) between socio-demographic variables and categorization of soiled cotton wool, swab, specimen container, body parts, fetuses, needles and scalpels. The respondents in the various facilities had adequate knowledge of waste categorization. 61.0 % indicated that segregation should be done at the source, as against 39.0 % who indicated otherwise and 88.6 % indicated the use of safety boxes for sharp collection. About 81.9 % of the respondents also indicated the need to segregate medical wastes. The responses however differed from hospital to hospital. 85.7 % of the respondents’ agreed that medical waste could be generated from diagnosis, immunization and treatment. About 74.3 % of the respondents also knew that there are specific procedures for collection and handling of medical waste (Table 4). There was no significant association (*p* ≥ 0.05) between socio-demographic variables and waste segregation. There was satisfactory knowledge of color coding of wastes which is an essential factor for proper segregation of waste. About 81.9 % of all the respondents indicated that they use color code for easy identification of the wastes generated in their various facilities. The majority of respondents also rightly identified the color codes of all the wastes generated. More than half of all the respondents (58.1 %) rightly identified the color code (black) for general waste, 53.3 % identify red as the color code for pathological waste but only 33.3 % of all the respondents could identify the color code for infectious waste as yellow (Table 5). There was a statistically significant association (*p* < 0.05) between the profession of the respondents and the ability to identify the color coding for pathological wastes with highest association amongst the nurses and this may be due to the training received.

**Table 3:** Assessment of Appropriate Waste Categorization by Respondents

Category of waste	Frequency	Percentage
Paper, Food, Plastic, Bottles		
Infectious waste	3	2.9
General waste	73	69.5
Pathological waste	9	8.6
Radioactive waste	2	1.9
Sharps	15	14.2
Pharmaceutical waste	3	2.9
Total	105	100
Soiled cotton wool, Swab, Gloves		
Infectious waste	73	69.5
General waste	12	11.4
Pathological waste	10	9.5
Radioactive waste	7	6.7
Sharps	1	1.0
Pharmaceutical waste	2	1.9
Total	105	100
Body parts, Body fluids, Fetuses		
Infectious waste	19	18.1
General waste	12	11.4
Pathological waste	61	58.1
Radioactive waste	4	3.8
Sharps	2	1.9
Pharmaceutical waste	7	6.7
Total	105	100
Needles, Scalpels, Syringes		
Infectious waste	2	1.9
General waste	3	2.9
Pathological waste	4	3.8
Radioactive waste	14	13.3
Sharps	73	69.5
Pharmaceutical waste	9	8.6
Total	105	100

**Table 4:** Generation and Segregation of Medical Wastes

Enquiry at each hospital	A	B	C	D	E	F	G
Should MW be segregated?							
Yes	10 (43.5)	39 (95.1)	9 (100.0)	4 (100.0)	10 (90.9)	7 (100.0)	7 (70.0)
I don’t know	-	2 (4.9)	-	-	1 (9.1)	-	2 (20.0)
Non response	13 (56.5)	-	-	-	-	-	1 (10.0)
Total	23 (100.0)	41 (100.0)	9 (100.0)	4 (100)	11 (100.0)	7 (100.0)	10 (100.0)
Can MW be generated during diagnosis, immunization, treatment							
Yes	18 (78.3)	35 (85.4)	9 (100.0)	3 (75.0)	10 (90.9)	7 (100.0)	8 (80.0)
I don’t know	-	2 (4.9)	-	1 (25.0)	1 (9.1)	-	2 (20.0)
Non response	5 (21.7)	4 (9.8)	-	-	-	-	1 (10.0)
Total	23 (100.0)	41 (100.0)	9 (100.0)	4 (100.0)	11 (100.0)	7 (100.0)	11 (100.0)
Do you have procedures for collection/handling of wastes							
Yes	12 (52.2)	35 (85.4)	9 (100.0)	1 (25.0)	9 (81.8)	6 (85.7)	6 (60.0)
No	7 (30.4)	-	-	3 (75.0)	-	-	1 (10.0)
I don’t know	1 (4.3)	3 (7.3)	-	-	2 (18.2)	-	2 (20.0)
Non response	3 (13.0)	3 (7.3)	-	-	-	1 (14.3)	1 (10.0)
Total	23 (100.0)	41 (100.0)	9 (100.0)	4 (100.0)	11 (100.0)	7 (100.0)	10 (100.0)
Location for MW segregation							
Source of generation	12 (52.2)	20 (48.8)	7 (77.8)	1 (25.0)	9 (81.8)	6 (85.7)	9 (90.0)
Outside the bin	7 (30.4)	14 (34.1)	2 (22.2)	-	1 (9.1)	1 (14.3)	-
I don’t know	-	3 (7.3)	-	3 (75.0)	1 (9.1)	-	-
Non response	4 (17.4)	4 (9.8)	-	-	-	-	1 (10.0)
Total	23 (100.0)	41 (100.0)	9 (100.0)	4 (100.0)	11 (100.0)	7 (100.0)	10 (100.0)
Is segregation done in operating theatre, labor rooms etc.							
Yes	4 (17.4)	26 (63.4)	7 (77.2)	-	9 (81.8)	5 (71.4)	7 (70.0)
No	3 (13.0)	14 (34.1)	2 (22.8)	-	2 (18.2)	2 (28.6)	-
I don’t know	-	1 (2.4)	-	-	-	-	2 (20.0)
Non response	16 (69.6)	-	-		-	-	1 (10.0)
Total	23 (100.0)	41 (100.0)	9 (100.0)	-	11 (100.0)	7 (100.0)	10 (100.0)
Type of container for sharps disposal.							
Nylon bag	6 (26.1)	-	-	-	-	-	-
Safety boxes	15 (65.2)	39 (95.1)	9 (100.0)	4 (100.0)	11 (100.0)	6 (85.7)	9 (90.0)
Non response	2 (8.7)	2 (4.9)	-	-	-	1 (14.3)	1 (10.0)
Total	23 (100.0)	41 (100.0)	9 (100.0)	4 (100.0)	11 (100.0)	7 (100.0)	10 (10.0)

**Table 5:** Color coding of medical waste

Enquiry at each hospital	A	B	C	D	E	F	G
Do you color-code your MW for disposal?							
Yes	13 (56.5)	37 (90.2)	9 (100.0)	-	11 (100.0)	7 (100.0)	9 (90.0)
No	7 (30.4)	-	-	-	-	-	-
I don’t know	1 (4.3)	2 (4.9)	-	-	-	-	-
Non response	2 (8.7)	2 (4.9)	-	4	-		1
Total	23 (100.0)	41 (100.0)	9 (100.0)	4 (100.0)	11 (100.0)	7 (100.0)	10 (100.0)
Color coding for PW.							
Red	12 (52.2)	18 (43.9)	6 (66.7)	-	9 (81.8)	5 (71.4)	6 (60.0)
Yellow	7 (30.4)	16 (39.0)	3 (33.3)	-	2 (18.2)	1 (14.3)	2 (20.0)
Brown	-	5 (12.2)	-	-	-	1 (14.3)	-
Yellow with radioactive symbol	-	2 (4.9)	-	-	-	-	
I don’t know	1 (4.35)		-		-	-	1 (10.0)
Non response	3 (13.0)		-	4 (100.0)	-	-	1 (10.0)
Total	23 (100.0)	41 (100.0)	9 (100.0)	4 (100.0)	11 (100.0)	7 100.0)	10 (100.0)
Color coding for IW							
Red	5 (21.7)	26 (63.4)	4 (44.4)	-	3 (27.3)	3 (42.9)	4 (40.0)
Yellow	5 (21.7)	11 (26.8)	5 (55.6)	-	6 (54.5)	4 (57.1)	4 (40.0)
Brown	2 (8.7)	-	-	-	-	-	-
Yellow with radioactive symbol	6 (26.1)	1 (2.4)	-	-	2 (18.2)	-	1 (10.0)
I don’t know	1 (4.3)	-	-	2 (50.0)	-	-	-
Non response	4 (17.4)	3 (7.3)	-	2 (50.0)	-	-	1 (10.0)
Total	23 (100.0)	41 (100.0)	9 (100.0)	4 (100.0)	11 (100.0)	7 (100.0)	10 (100.0)
Color code for GW							
Red	1 (4.3)	-	-	-	-	-	-
Brown	2 (8.7)	8 (19.5)	2 (22.2)	-	-	2 (28.6)	-
Black	15 (65.2)	17 (41.5)	7 (77.8)	4	8 (72.7)	3 (42.9)	7 (70.0)
Non response	5 (21.7)	16 (39.0)	-	-	3 (27.3)	2 (28.6)	3 (30.0)
Total	23 (100.0)	41 (100.0)	9 (100.0)	4 (100.0)	11 (100.0)	7 (100.0)	y100.0)

*MW* medical waste, *PW* pathological waste, *IW* infectious waste, *GW* general waste

The result indicates that various means of on-site transportation of waste from the source of generation are utilized with wheel barrows and trolleys constituting the major means of evacuating the waste. Although, facility B has a hospital constructed truck for the same purpose.

It was likewise observed during the visits that all the surveyed hospitals outsource their waste to LAWMA medical. The treatment of waste within the hospitals is not common except for one of the public facilities (B) which uses incinerator to treat its sharp. This hospital also engages the services of an environmental officer who oversees the treatment and eventual disposal of its medical wastes. The majority of respondents are now aware that LAWMA MEDICAL is in charge of medical waste in Lagos State.

## Discussion

The majority of the respondents were domestic workers. The aforementioned is in contrary with the study of Joshua et al. [26] which was carried out in some primary health care centers in Zaria - Nigeria where majority (37 %) were nurses and no domestic workers were used for the survey on waste disposal and management. The involvement of the domestic workers in waste management is inevitable and logical as they are largely involved in waste collection and transportation.

It is quite clear that for efficient waste management program the quantity and variations in the waste generated in each facility must be put into considerations. The findings in this study corroborate some rates recorded in Souss-Massa-Draa, where an average rate of 0.53 kg/bed/day was recorded [1]. Furthermore, a study carried out in 2008 by Abdulla et al., showed that waste weighted average was 0.83 kg/bed/day in northern Jordan and 1.22 kg/bed/day was reported by Ruoyan et al., in 2010 as weighted average rate in Binzhou Distrinct in China [27, 28]. The earlier study done by Longe and Williams, in Lagos State before the introduction of MWM, reported an average generation rate of 0.573 kg/bed/day [25]. The reduction that was noted in this study for the average generation rate may be attributed to the intervention of Lagos State, through the awareness and training programs organized by LAWMA medical unit for proper segregation of infectious waste, adequate categorization and disposal of the waste.

Wastes generated from the various activities performed in hospitals include general and medical wastes. The general waste emanates from food preparation, administrative activities, landscaping, housekeeping, activities of health-care establishments and may also include waste generated during maintenance of health-care premises. This type of waste may be similar to household and city wastes.

While the wastes generated in the health facilities include cultures, stocks of infectious agents, pathological, blood and other fluids, sharps, surgery and laboratory wastes, wastes from food preparation, radioactive wastes, wastes from dialysis procedures, biological wastes, cardboard, paper documents and discarded linens. Between 75 and 90 % of the waste produced by health-care facilities is non-risk or general health-care waste, which is comparable to domestic waste, while about only 25 % is regarded as hazardous and may create a variety of health risks [14].

Waste generation source, categorization, quantity and quality are the key issues to decide an effective medical waste management practice [1]. The medical staff in the surveyed hospitals had adequate knowledge of the various categories of the wastes generated.

Two-third of all the respondents rightly categorizes both the general and infectious waste which thus leads to proper segregation of the waste. A further analysis indicates that higher number of nurses rightly identified items that constitute MW more than other profession. The justification for this observation was witnessed during the in-depth interview section, where nurses displayed higher knowledge about the medical waste categorization than others. This is due to the fact that they go for more training, both in-house and those organized outside their facilities on hospital waste management and also with the inclusion of the capacity building sessions annually organized by Lagos waste management authority (LAWMA).

In general, respondents are aware of the fact that medical waste can be generated during immunization, treatment, diagnosis, medical research, given the high proportion of respondents who provided the right answer to an enquiry on this issue.

Segregation of infectious waste at the source of generation is the key to achieving a sound medical waste management. The study revealed that majority of respondents agreed on segregation of medical waste at the point/source of generation. This is consistent with the findings of Asadullah, et al. [29] which indicated that 90.4 % of respondents were of the view that segregation of waste should be at the point of generation. It is important to note that medical waste segregation is an important step in reducing the volume of hazardous waste. Such segregation is achieved by making use of labeled containers or colored liners to effectively separate infectious waste from general/domestic waste. More than three quarters of the respondents uses safety boxes for sharp collections and this is in accordance with the regulation of WHO which ensures that the sharps are properly secured and do not fall out of the container and it should only be three-quarters filled prior to disposal [30].

The high percentage of respondents using color code for identification indicates their level of understanding its essence in management of medical waste. It also helps with easy recognition and disposal of the waste. This is also consistent with the findings of Abdullah and Al- Mukhtar in 2013 where about 79.2 % of the respondents uses color coding for proper identification but contrary views was noted in the findings done in 2005 by Al-Khatib and in Zaria by Joshua et al. [26], where none of the facilities practice color coding for segregation and thus reflected in their practices [31, 32].

There was satisfactory knowledge of color coding of wastes which is an essential factor for the proper segregation of waste. Proper segregation is achieved by making use of actual colored containers or colored liners to effectively separate infectious waste from general/domestic waste. WHO [30], proposed that hospitals should provide either plastic bags or strong plastic containers for medical wastes and that they should make use of different colored liners namely, Black, Yellow and Red (three bin system) for general, infectious and highly infectious waste respectively. Bags and containers for highly infectious waste should be marked with Biohazard symbol [33]. The use of a brown liner is also encouraged by WHO for pharmaceutical waste (expired drugs) but this is rarely used. There was a statistically significant association between the profession of the respondents and the ability to identify the color coding for pathological wastes with highest association amongst the nurses and this is also due to the training received.

Various means were utilized to transport wastes from the point of generation to the on-site storage; while wheel barrows and trolleys constituted the major means of evacuating wastes in most facilities which is similar to the findings by Joshua, et al. [26], however, only facility B used hospital constructed trucks. Medical wastes generated in hospitals are collected on a daily basis and transported to a temporary storage center within the hospital.

Such wastes are collected and transported by the means of a trolley, wheeled barrow, trucks etc. Data from this study revealed that one of the two public hospitals (hospital B) uses trucks (hospital constructed), while some use trolley and others conveys the waste by hand which could be dangerous. Although WHO stipulates that different trolleys should be used in transporting the different categories of wastes, this requirement is not adhered to in most hospitals that were surveyed. Indeed, all the wastes generated are carried with the same trolley and this could also lead to cross-contamination. Domestic staff/sanitary officers are responsible for collection of the segregated medical wastes from the wards to the on-site storage center in all the hospitals. As important as protective equipment are to anybody who handles medical wastes, the hospitals surveyed use only heavy duty gloves and this is not consistent with the recommended standard of WHO which requires the use of heavy duty gloves, boots and apron [33]. A study which was carried out in Tehran University by Dehghani et al. [3] indicated the compliance with WHO standard by using the complete personal protective wear. Safety shoes or industrial boots should also be encouraged because they help to protect the feet against the risk of sharp being accidentally dropped, thereby causing a prick. There is need to properly equip and educate those in charge of on-site transportation of wastes, given the great danger associated with this task. The use of adequate and complete protective clothing is very vital.

Medical waste treatment leads to a reduction in volume, weight and risk of infection and organic compound of the waste [33]. There are no clear policies and plans in place for managing medical waste in the surveyed hospitals, as evidenced by the absence of manuals and guidelines. On further enquiry, it was discovered that even the Ministry of Health does not have manuals or guidelines for the management of hospital wastes. Indeed, it was gathered that there is no medical waste management policy/guideline at both the national and state levels. It is important for Standard Operating Procedure (SOP) to be prepared for medical waste management in the hospitals as obtained in developed countries where definite rules and regulations exist at the national, regional and hospital levels. In the light of this it is not only the policy/legislation but also the inclusion of proper monitoring and enforcement strategy, which would further allow for proper MWM [9]. The study also noticed several reasons for poor HCWM in the hospitals but the most prevalent challenges highlighted during the interview section were lack of definite policies/legislation, lack of budget allocation, lack of rules and regulations, poor training of some hospital staff and lack of implementation/enforcement.

## Conclusion

Despite the challenges associated with WM especially the lack of policies and regulations as stipulated by WHO. Lagos state has taken the initiatives to have a well-organized system of collecting and treating waste. The State has also taken further steps by providing the needed items like the different colored containers, liners to the hospitals at no cost. LAWMA also collects the waste for final disposal at little cost so that the hospitals can be encouraged to segregate and collect their waste appropriately. From the findings of this study, it suffices to conclude that there is little progress in the management of medical waste in Lagos State because of the following: The MWM practices among the various hospitals surveyed are similar except for hospital D which still mixes its medical and general waste. The medical waste is collected and segregated using the three colors coding system by WHO, then transfer to the on-site storage and finally transported by Lagos State to the transfer loading station where it is treated by means of hydroclave. This system is congruence with WHO specifications however; uniformity in MWM practices should be ensured in all hospitals as against the divergent of hospital D. The level of awareness and training among the workers has relatively increased due to the intervention of LAWMA and John Snow Inc. however; continuous training of the hospital staff on MWM is highly advocated. There is also a need for awareness of waste management system amongst the patient/community in order to prevent nosocomial infections and environmental hazards. Policy and regulation guidelines should be provided to all the three tiers of government (federal, state and local government) so as to improve waste management practices throughout the country as also recommended in South Africa by Pululu and Tabukeli [34].

## Abbreviations

HBC: hepatitis C virus
HBV: hepatitis B virus
HCF: healthcare facility
HEFEMA: Health Facility Monitoring and Accreditation Agency
LAWMA: Lagos Waste Management Authority
MW: medical waste
MWM: Medical Waste Management
WHO: World Health Organization
